# A novel method for DNA delivery into bacteria using cationic copolymers

**DOI:** 10.1590/1414-431X202010743

**Published:** 2021-04-02

**Authors:** V.V. de Souza, P.A.M. Vitale, F.H. Florenzano, R.K. Salinas, I.M. Cuccovia

**Affiliations:** 1 Departamento de Bioquímica, Instituto de Química, Universidade de São Paulo, São Paulo, SP, Brasil; 2 Departamento de Engenharia de Materiais, Escola de Engenharia de Lorena, Universidade de São Paulo, Lorena, SP, Brasil

**Keywords:** Gene delivery, Bacterial transformation, Copolymers, DMAEMA

## Abstract

Amphiphilic copolymers have a wide variety of medical and biotechnological applications, including DNA transfection in eukaryotic cells. Still, no polymer-primed transfection of prokaryotic cells has been described. The reversible addition-fragmentation chain transfer (RAFT) polymer synthesis technique and the reversible deactivation radical polymerization variants allow the design of polymers with well-controlled molar mass, morphology, and hydrophilicity/hydrophobicity ratios. RAFT was used to synthesize two amphiphilic copolymers containing different ratios of the amphiphilic poly[2-(dimethyl-amino) ethyl methacrylate] and the hydrophobic poly [methyl methacrylate]. These copolymers bound to pUC-19 DNA and successfully transfected non-competent *Escherichia coli* DH5α, with transformation efficiency in the range of 10^3^ colony-forming units per µg of plasmid DNA. These results demonstrate prokaryote transformation using polymers with controlled amphiphilic/hydrophobic ratios.

## Introduction

Amphiphilic copolymers self-assemble in water, forming various aggregates that exhibit a wide variety of technological applications (1,2). Amphiphilic copolymers may respond to diverse stimuli such as temperature, pH, or ions (3-6). Modulation of polymer solubility by pH and temperature provides an efficient way to control the delivery and release of trapped molecules (7,8). Amphiphilic cationic copolymers bind to DNA, facilitating eukaryotic cell transformation, thereby constituting an alternative to viral DNA/RNA vectors (9,10). Cationic polymers synthesized from 2-(dimethyl-amino) ethyl methacrylate (DMAEMA) with different morphologies facilitate DNA delivery to eukaryotic cells but are cytotoxic (11-13). Other polymeric materials deliver DNA into eukaryotic cells, but a polymeric system capable of transfecting bacteria has not been described. Copolymer morphology and composition may be modulated to maximize gene delivery efficiency and, hopefully, minimize toxicity. Gradient or random copolymers of poly [DMAEMA] (PDMAEMA) with few polar units could have advantages over previous DMAEMA-based copolymers tested for DNA delivery (14). Systematic changes of copolymer molar and composition ratios may permit the design of better materials for DNA delivery, eventually with reduced toxicity (15).

**Figure 1:**
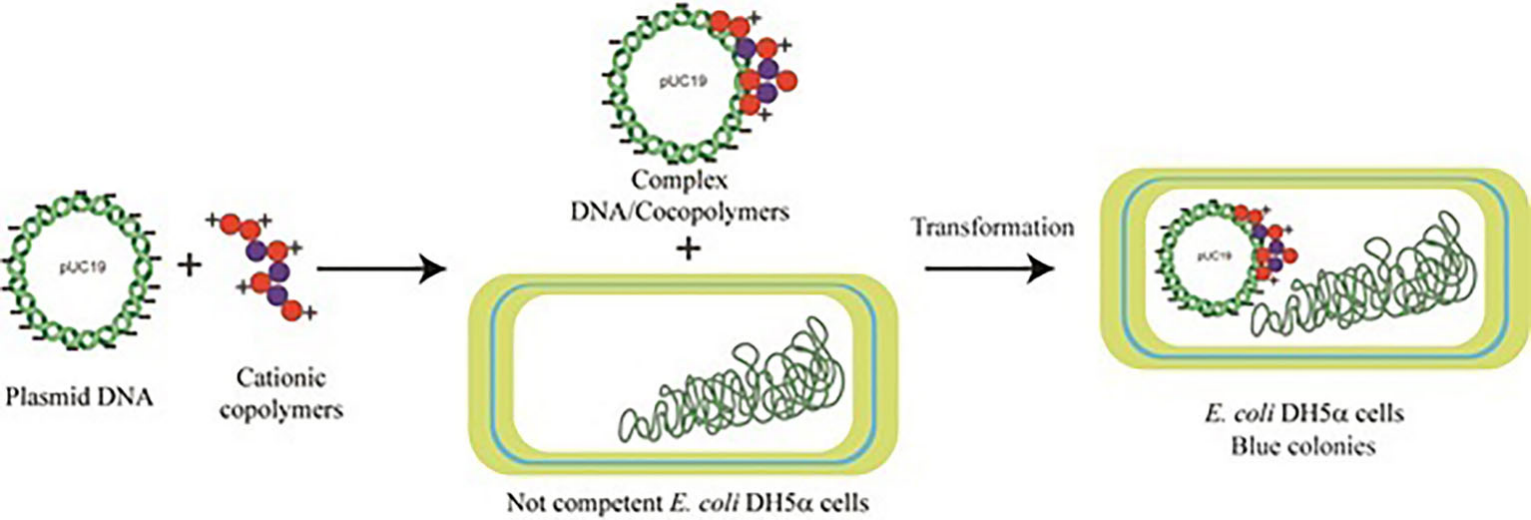
PDMAEMA-based cationic copolymers as novel carriers for DNA delivery into bacteria. Here we show that amphiphilic copolymers containing DMAEMA deliver DNA into *E. coli* cells opening the way to the development of new DNA delivery agents.

Here, we synthesized positively charged polymeric materials as potential DNA vectors using synthetic methods, including free radical polymerization (FRP) (16,17), reversible addition-fragmentation chain transfer polymerization (RAFT), and a reversible deactivation radical polymerization. Polymers consisting of PDMAEMA and poly (methyl methacrylate) (PMMA) obtained via FRP or RAFT bind DNA and efficiently promoted plasmid DNA transfer into *Escherichia coli*. These observations pave the way towards developing new synthetic materials based on PDMAEMA-co-PMMA copolymers, with optimized properties to carry DNA into eukaryotic and prokaryotic cells (Figure 1).

## Material and Methods

### Materials

Methanol, n-hexane, acetone (analytical grade), and triethylamine (TEA) (HPLC grade) were from J.T. Baker (USA). Tetrahydrofuran (THF), deuterated chloroform (CDCl_3_), methyl methacrylate (MMA, 99%), and dimethyl 2-(aminoethyl) methacrylate (DMAEMA, 98%) were from Sigma-Aldrich (USA) and their polymerization inhibitor, MEHQ, was removed using De-HiBit-200 (Polysciences, Inc., USA) macroreticular ion exchange resin. 2,2′-azobisisobutyronitrile (AIBN, 98%), 1,1′-azobis(cyclohexane carbonitrile) (ACHN, 98%), 2-cyano-2-propyl dodecyl trithiocarbonate (chain transfer agent, CTA), and buffers were from Sigma-Aldrich. NaH_2_PO_4_, boric acid, HCl, and NaOH were from Merck (Germany). Isopropyl β-D-1-thiogalactopyranoside (IPTG) and 5-bromo-4-chloro-3-indolyl β-D-galactoside (X-Gal) were from Thermo Scientific (USA). Gel permeation chromatography (GPC) standard for average molar mass determination was Poly(methyl methacrylate) Standard ReadyCal Set, Mp 500-2,700,000, from Sigma Aldrich.

### Chemical syntheses

All syntheses were carried out under argon and constant stirring at 600 rpm. The growth of the polymer chains during synthesis was monitored by GPC analysis of reaction aliquots taken at increasing reaction times.

### Free radical polymerization

PMMA-co-PDMAEMA was synthesized via FRP technique using ACHN as initiator. PMMA_3_-co-PDMAEMA_26_ (FRP): 5 mL (46.7 mmol/L) of MMA, 20 mL (118.7 mmol/L) of DMAEMA, and 0.289 g (1.18 mmol/L) of ACHN were mixed in a reaction flask with 10 mL of 1,4-dioxane. The polymerization reaction was carried out for 35 min at 90°C under constant stirring. The copolymer was purified by repeated precipitation into hexane, which was oven-dried at 40°C for 48 h.

### RAFT polymerizations

PMMA and PDMAEMA homopolymers and PMMA-co-PDMAEMA were synthesized via RAFT technique using 2-cyano-2-propyl-dodecyl trithiocarbonate as the chain transfer agent (CTA), chosen considering the reactivity and compatibility with MMA and DMAEMA. The CTA used in all RAFT syntheses described herein is commonly used for polymerizing styrenes, methacrylates, and methacrylamides (17). The PDMAEMA synthesis was initiated using 1,1′-azobis(cyclohexanecarbonitrile) and all other reactions were initiated by azobisisobutyronitrile (AIBN). All syntheses were controlled with 2-cyano-2-propyl dodecyl trithiocarbonate as the CTA.

#### PDMAEMA_315_

Initially, 100 mL (0.59 mol/L) of DMAEMA, followed by the addition of 0.2154 g (0.623 mmol/L) of the CTA and 0.038 g (0.155 mmol/L) of ACHN were mixed in the reaction flask. The reaction was carried out at 85°C for 5 h. The homopolymer product was purified by repeated precipitation in hexane and, subsequently, dried in an oven for 48 h at 40°C.

#### PMMA_60_

Twenty milliliters (0.19 mmol/L) of MMA monomer was dissolved in 10 mL of 1,4-dioxane, followed by the addition of 0.45 g (1.30 mmo/L) of CTA and 0.0355 g (0.21 mmol/L) of AIBN. Synthesis was carried out under constant stirring for 5 h at 70°C. Purification was performed by repeated precipitations in methanol. The resulting solid was dried in an oven for 48 h at 40°C.

#### PMMA_31_-co-PDMAEMA_70_

Twenty milliters (0.186 mol/L) of MMA and 75 mL (0.444 mmol/L) of DMAEMA monomer, free of polymerization stabilizer, 1.314 g (3.8 mmol/L) of CTA and 0.104 g (0.633 mmol/L) of AIBN were mixed in a reaction flask under argon atmosphere for 3 h at 70°C. GPC was used to monitor the synthesis progress. The reaction was stopped after the desired molecular weight (M_n_: 14,000 g/mol) was reached. The final product was purified by repeated precipitations in hexane and subsequently dried at 40°C for 96 h.

#### GPC

**Figure 2:**
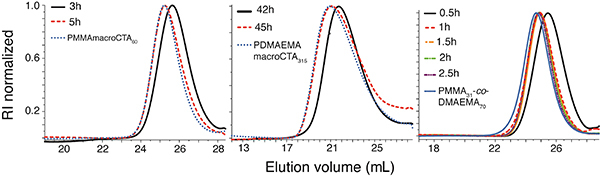
Polymer growth with synthesis time. Gel permeation chromatography traces showing the growth of homopolymer and copolymer chains by reversible addition-fragmentation chain transfer over time. PMMA_60_ (**left**); PDMAEMA_315_ (**middle**); PMMA_31_-co-PDMAEMA_70_ (**right**). The time course of the reaction is within each panel. RI: refractive index.

GPC was performed in a Shimadzu Prominence instrument (Japan) equipped with two Phenomenex (USA) columns (particle size: 5 μm, pore sizes: 10^6^ Å and 10^4^ Å). The injected sample volume (10 mg/mL) was 10 µL. Column temperature was kept at 35°C. Detection was based on differential refractive index (RI) (Shimadzu RID-10A, Japan). The mobile phase was THF with 0.3 % of TEA as eluent at a flow rate of 0.8 mL/min. The system was calibrated with PMMA standards (M_n_ ∼ 800−2,000,000 g/mol) (Figure 2).

### Nuclear magnetic resonance (NMR) spectroscopy

**Figure 3:**
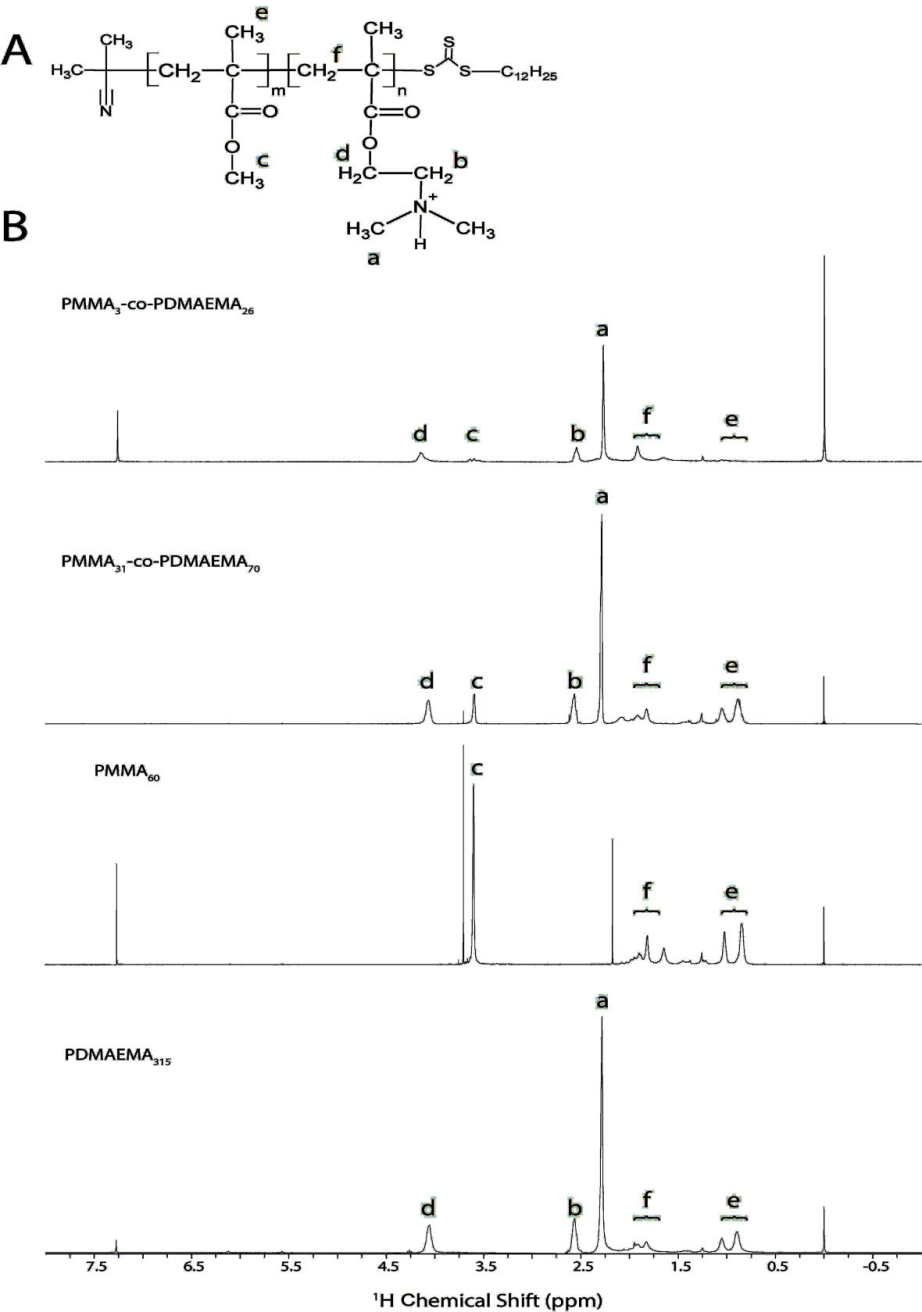
Structure and nuclear magnetic resonance (NMR) spectra of the polymers. General chemical structures (**A**) and (^1^H) NMR spectra (**B**) of PMMA-co-PDMAEMA copolymers. The letters “m” and “n” refer to the number of MMA and DMAEMA units, respectively. All reversible addition-fragmentation chain transfer copolymers are expected to have chain-transfer agent terminations. The (^1^H) NMR peaks at 4.05 and 3.60 ppm correspond to the (O-CH_2_-) of DMAEMA and the (O-CH_3_) of MMA, respectively.

One dimensional ^1^H-NMR spectra were recorded at room temperature on a Varian 300 MHz NMR spectrometer (USA). NMR samples consisted of 10 mg of polymer dissolved in CDCl_3_. PMMA and PDMAEMA ^1^H chemical shift assignments were taken from the literature (18-21). PDMAEMA and PMMA ratios in the different copolymers (n/m ratios) were calculated from the relative areas under the ^1^H NMR peaks corresponding to the side chain methylene group of the ester of PDMAEMA at approximately 4.0 ppm (2H, O-CH_2_-, PDMAEMA, see Figure 3) and to the ester methyl group of MMA at approximately 3.6 ppm (3H, O-CH_3_, PMMA), according to Equation 1:

$$\frac{{\text{n}}_{\left(\text{PDMAEMA}\right)}}{{\text{m}}_{\left(\text{PMMA}\right)}}\;=\;\frac{{\text{A}}_{\text{d}}\;\text{x}\;3}{{\text{A}}_{\text{c}}\;\text{x}\;2}$$ (Eq. 1)

where A_d_ and A_c_ refer to the area under the peak of the PDMAEMA methylene group and the PMMA methyl group, respectively. The coefficients 2 and 3 normalize the areas with respect to the number of hydrogen nuclei contributing to each NMR signal, while n and m are the number of units of DMAEMA and MMA, respectively. The total polymer mass, M_n_, is given by the relative composition of the two monomers as described in Equation 2:

$${\text{M}}_{\text{n}}=\;\text{n*}\left(\text{PDMAEMA}\right)+\;\text{m*}\left(\text{PMMA}\right)\;$$ (Eq. 2)

where (PDMAEMA) and (PMMA) are the monomer molecular masses, i.e., 157.9 and 100, respectively.

### Propagation and purification of pUC19

Transformation of competent *E. coli* DH5α was carried out following standard methods (22-25). Briefly, approximately 50 ng of pUC19 were mixed with 50 µL of chemically competent DH5α on ice for 30 min. The mixture was subjected to heat shock at 42°C for 45 s, incubated on ice for 1 min, followed by the addition of 750 μL of liquid sterile Luria broth (LB) (Sigma, USA) for cell growth at 37°C for 1 h. Cells were harvested by centrifugation at 8,000 *g* for 5 min at room temperature, suspended with 100 μL of LB, and plated on LB agar containing 100 µg/mL of ampicillin, 1 mmol/L of IPTG and 20 µg/mL of X-Gal. Bacterial cells were grown on plates overnight at 37°C. On the next day, one colony of bacteria harboring pUC19 was selected based on the blue-white screening test and incubated in liquid LB under agitation at 230 rpm and 37°C for overnight growth. pUC19 plasmids were isolated using the Plasmid Plus Maxi kit (Qiagen, USA) according to the manufacturer instructions (26,27).

### Electrophoretic assays

Agarose gel electrophoretic assays were performed using 1% agarose gels in TAE (Tris-Acetate-EDTA) buffer prepared with 6 µL of SYBR Safe (Invitrogen, USA). The desired aliquots (30, 50, and 100 µL) of a stock solution of each copolymer (1 mg/mL in THF) were transferred to plastic microtubes, and the solvent was evaporated under argon to allow the formation of polymeric films. A volume of 18 µL of a water solution of pUC19 DNA at a concentration of 78 ng/µL (determined based on the absorbance at 260 nm) was added, followed by vortexing for 10 s, and resting on ice for 5 min. An aliquot of 6 µL of DNA loading buffer (Purple 6x) (Biolabs, Inc., USA) was added to each tube, which was again vortexed for 10 s. All samples were maintained on ice in order to preserve the DNA from degradation. Electrophoresis was run in TAE buffer using 50 volts.

### Transformation of *E. coli* with polymer/pDNA polyplexes

In order to verify the influence of the amount of each polymer on the bacteria transformation efficiency, desired aliquots (0.25, 0.5, 1.0, and 5.0 µL) of polymers taken from a 1 mg/mL stock in THF were transferred to plastic microtubes, followed by evaporation of the THF under argon until the formation of a film. Non-competent *E. coli* DH5α cells were cultured in 100 mL of LB (Sigma) without antibiotics until the absorbance reached 0.7 (λ=600 nm). Cells were harvested by centrifugation (8,000 *g*, for 5 min at 4°C), suspended into 500 µL of 10 mM MOPS pH 6.8, and reserved on ice. An aliquot of 10 µL of a 78.7 ng/µL of pUC19 DNA solution in water was added to hydrate the polymeric film in the plastic microtube, followed by vigorous vortexing for one minute. After vortexing, the mixture rested for 5 min at 37°C. A volume of 700 µL of LB was added to the hydrated polymeric film, followed by the addition of 100 µL of non-competent DH5α prepared as mentioned above, vortexed for 1 min, and incubated at 37°C for 1 h. A volume of 50 µL of DH5α cells incubated with polymer and pUC19 (49 ng) were plated on LB-agar prepared with 100 µg/mL of ampicillin, 20 µg/mL of X-Gal, and 1 mmol/L of IPTG, and incubated at 37°C for 36 h.

## Results and Discussion

Although PDMAEMA homopolymers bind to DNA (28,29), PDMAEMA-mediated DNA transfer to bacterial cells has not been reported. PDMAEMA binding to DNA results from electrostatic interactions but bacterial transfection may depend on the hydrophobic/hydrophilic balance of the polymeric material. To test whether increasing hydrophobicity could raise the ability of the polymer to mediate DNA transfer into bacterial cells, we designed amphipathic copolymers of different sizes and compositions. Homopolymers of PMMA, PDMAEMA, and copolymers containing DMAEMA/MMA units were synthesized to determine the impact of the different DMAEMA/MMA ratios on bacterial transformation efficiency. MMA was chosen as the hydrophobic component because it has been used to synthesize biocompatible materials for a wide variety of applications (30,31).

PMMA-co-PDMAEMA copolymers were synthesized either via free radical polymerization or via RAFT (see Methods), while PDMAEMA and PMMA homopolymers were synthesized via RAFT. Figure 2 shows GPC traces for all materials produced by RAFT. For this type of polymerization, average molar mass (M_n_) increase with conversion is expected (17). Average molar mass growth is detected by the displacement of the elution peak to lower volumes as a function of reaction time (32). After reaching the desired molecular weight (M_w_), the reaction was stopped, and the final product was purified.

^1^H-NMR of all copolymers (Figure 3) agreed with the literature (33). The peaks attributed to the main chain protons (“e” and “f”, Figure 3) were within the 0.7-2.0 ppm range. Peaks assigned to the PDMAEMA units were at 2.30 ppm (“a”, N-CH_3_), 2.58 ppm (“b”, N-CH_2_-), and 4.08 ppm (“d”, O-CH_2_-), respectively. The peak at 3.60 ppm was assigned to the PMMA side chain protons (“c”, O-CH_3_). The composition of the synthesized products, determined by ^1^H NMR (Figure 3) as described in Methods section, using the areas of the signals “c” and “d”, are presented in Table 1. As the total average molar masses were determined by GPC using PMMA standards, the molar mass units refer to PMMA molar mass / hydrodynamic ratio relationships. The polymers obtained by RAFT presented low polydispersity (M_w_/M_n_) characteristic of this technique (Table 1) (17).

**Table 1:** Synthesis parameters and selected characterization data.

Material	Method	M_n_ (kg/mol)	M_w_/M_n_
PMMA_60_	RAFT	12.4	1.3
PDMAEMA_315_	RAFT	54.6	1.4
(a) PMMA_3_-co-PDMAEMA_26_	FRP	4.45	1.9
(b) PMMA_31_-co-PDMAEMA_70_	RAFT	14.0	1.3

M_n_ and M_w_/M_n_ were measured by gel permeation chromatography. RAFT: reversible addition-fragmentation chain transfer; FRP: free radical polymerization.

**Figure 4:**
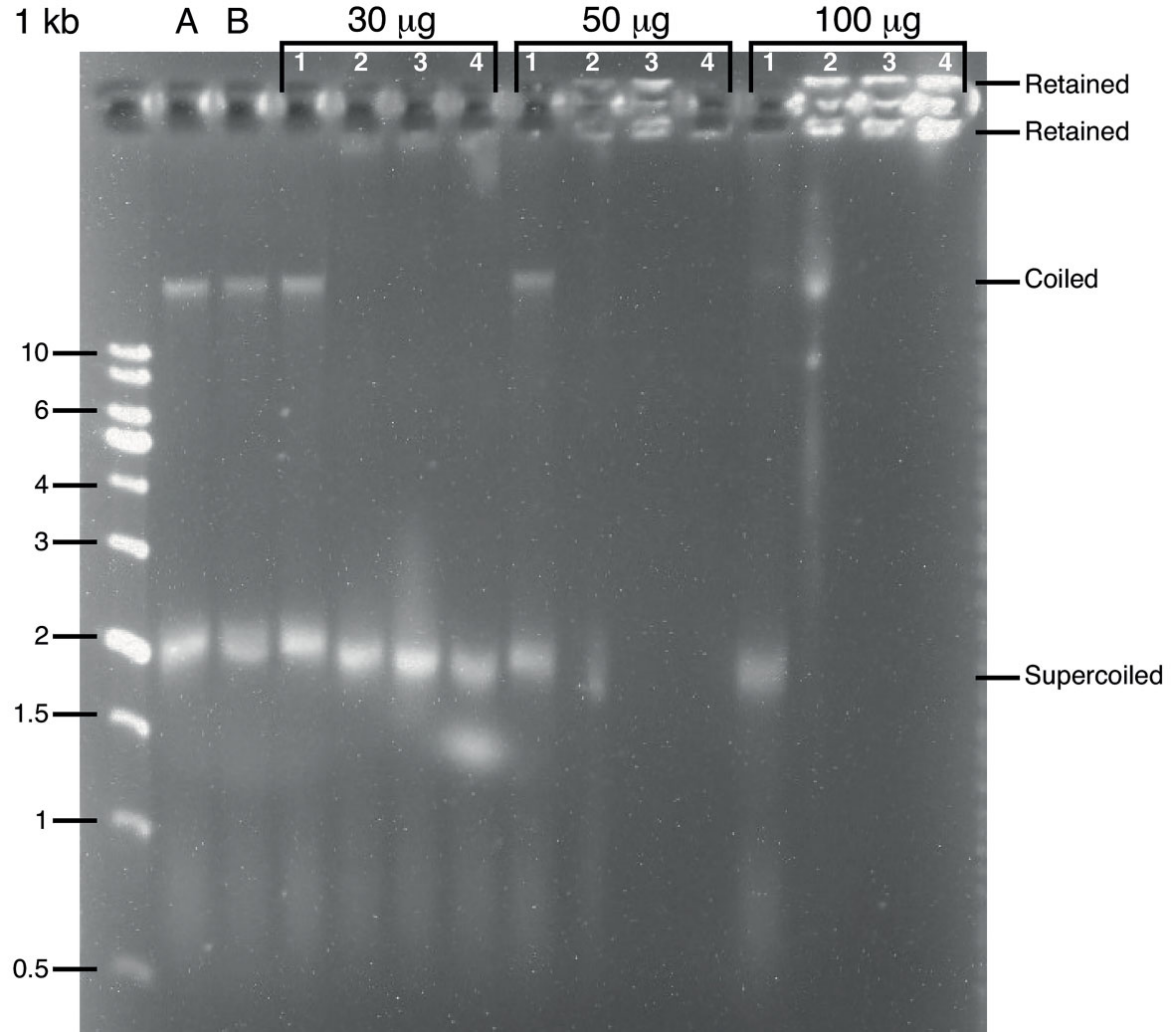
DNA polymer binding. Copolymer binding to pUC19 shifts its migration band on agarose gel. The agarose gel electrophoresis was run for 90 min at 50 volts. The following amounts of copolymers were tested: 30, 50, and 100 µg. Lanes A and B refer to controls corresponding to DNA only in the presence of tetrahydrofuran (A) or buffer (B) added in substitution of copolymer. The copolymers added in each experiment are indicated by numbers: 1 = PMMA_60_; 2 = PDMAEMA_315_; 3 = PMMA_3_-co-PDMAEMA_26_ (FRP); 4 = PMMA_31_-co-PDMAEMA_70_.

Agarose gel electrophoresis of pUC19 demonstrated that DMAEMA-containing copolymers bind to DNA (Figure 4). pUC19 is a high-copy standard cloning vector containing the coding sequence of the alpha fragment of beta-galactosidase (*lacZ*). pUC19 electrophoretic migration in the agarose gel yielded two bands, one of them corresponding to circular DNA, migrating with an apparent size larger than 10 kb, and supercoiled DNA that migrated with an apparent size near 2 kb (Figure 4). This behavior is consistent with uncut plasmid DNA. Addition of either PDMAEMA_315_ or PMMA_3_-co-PDMAEMA_26_ (FRP) and PMMA_31_-co-PDMAEMA_70_ led to altered pUC19 migration and even retention of DNA near the application slot. PMMA_60_, on the other hand, did not affect pUC19 migration. These results confirmed the ability of PDMAEMA-containing polymers to bind to plasmid DNA. DNA binding is related to the presence of PDMAEMA, since methyl polymethacrylate (PMMA_60_) at concentrations of 30 and 50 µg did not affect DNA migration (Figure 4). Clearly, PDMAEMA monomers bearing a net positive charge are required for DNA binding and electrophoretic mobility shifts.

**Figure 5:**
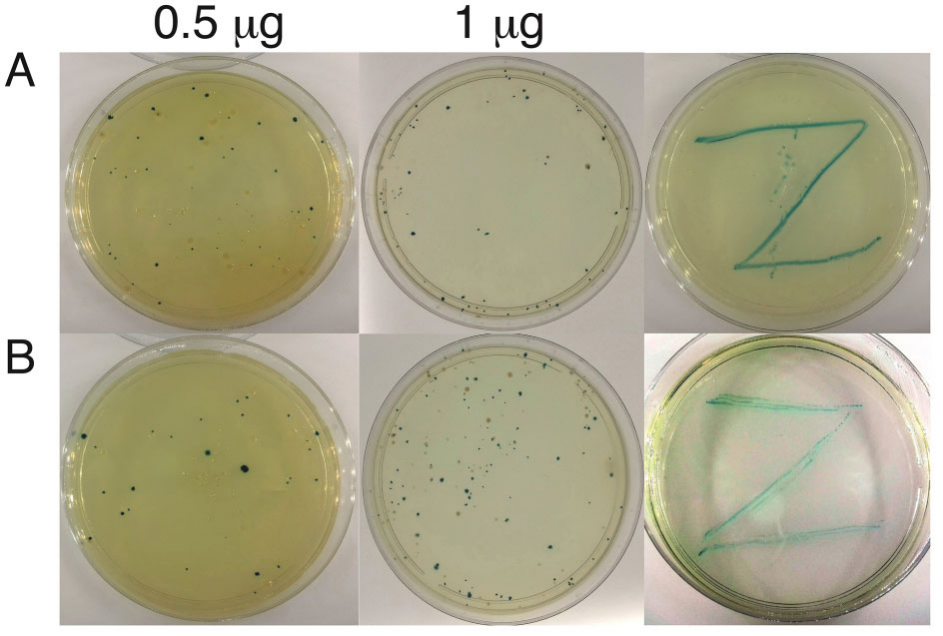
Demonstration of pUC19 transfection by polymers. Expression of pUC19 in *E. coli* DH5α using the blue-white screening test. **A**, PMMA_3_-co-PDMAEMA_26_ (FRP). **B**, PMMA_31_-co-PDMAEMA_70_. The amount of copolymer used in these experiments was 0.5 µg (left plates) and 1 µg (middle plates). The plates on the right refer to the inoculation of a single bluish colony, from the plates incubated with 1 µg of polymer, in new agar plates containing IPTG and X-Gal. FRP: free radical polymerization.

After observing that PDMAEMA-containing copolymers bind to plasmid DNA, we investigated whether these were able to mediate pUC19 delivery to non-competent *E. coli* cells, i.e., transfect. Bacterial colonies harboring pUC19 may be identified by the blue-white screening test. The expression of the *lacZ* fragment upon induction with IPTG results in a functional β-galactosidase that catalyzes the cleavage of the glycosidic bond in the chromogenic substrate X-Gal added to the agar plates, yielding a blue pigment and hence blue colored colonies (34). Bacteria transformed with functional pUC19 were identified as blue colonies on agar plates (Figure 5). We found that only those *E. coli* cells that were incubated with pUC19 in the presence of PMMA_3_-co-PDMAEMA_26_ (FRP) or PMMA_31_-co-PDMAEMA_70_, but not in the presence of PDMAEMA_315_ or PMMA_60_ (not shown), yielded blue colored colonies in the agar plates. Controls performed in the absence of the polymer, which was substituted for THF or buffer, displayed no colonies (not shown). A single blue colony from the previous experiment was plated in a new LB-agar plate containing IPTG and X-Gal, yielding additional blue colonies after 12 h of incubation at 37°C (Figure 5). Therefore, the transformed colonies were fully functional, retaining the ability to grow and express the *lacZ* fragment.

*E. coli* cell growth was slower in the presence of copolymer. After transformation in the presence of copolymers, new colonies were visually observed on plates only after 36 h of incubation at 37°C instead of the usual growth time of 12 h. In order to quantify the copolymer transformation efficiency, we counted the number of CFU (colony forming units) per microgram of DNA (Table 2). Copolymer transformation efficiency was in the range of 10^3^ CFU/µg of plasmid DNA and depended on the amount of copolymer used. The best copolymer amount was 0.5 µg under our conditions (Table 2). We did not observe blue colonies when *E. coli* was transfected in the presence of PMMA_60_, in line with the observation that this polymer does not bind to DNA (Figure 4). Interestingly, PDMAEMA_315_ homopolymer also failed to transform *E. coli* even though it binds to DNA (Figure 4). The observed transformation efficiency, in the range of 10^3^ CFU/µg of plasmid DNA (Table 2), was lower than that usually obtained with chemically competent *E. coli* cells using calcium chloride (approximately 5-20 10^6^ CFU/µg). However, various factors may affect transformation efficiency, including the amount of viable cells. The number of colonies obtained displayed large variation between different transformation trials (Table 2). Although this last observation may be seen as a disadvantage of the method, the present results should be seen as a proof of principle. The contributions of all variables remain to be investigated in the future. In fact, one should keep in mind that fine-tuning the morphology of the copolymers could result in more efficient transfecting agents.

**Table 2:** *Escherichia coli* transformation efficiency (10^3^ CFU/µg of DNA).

Polymer	Transformation efficiency^a^			
	0.25 µg^b^	0.5 µg^b^	1 µg^b^	5 µg^b^
PDMAEMA_315_	0	0	0	0
PMMA_60_	0	0	0	0
PMMA_3_-co-PDMAEMA_26_ (FRP)	0.20±0.04	1.08±0.09	0.63±0.52	0.02±0.01
PMMA_31_-co-PDMAEMA_70_ (RAFT)	0.08±0.08	0.88±0.12	0.98±0.47	0
THF	0	0	0	0
Buffer	0	0	0	0
Heat shock^a^	0	0	0	0

CFU: colony forming units; ^a^non-competent cells; ^b^amount of polymer added (from 1 mg/mL of tetrahydrofuran (THF) solution). Data are reported as means±SD of three experiments.

The presence of a small fraction of PMMA in the polymer chains was necessary for transfection. Note that only one homo PDMAEMA was used and that polymer was obtained exclusively by RAFT. A systematic change of those parameters is still needed in order to determine if PDMAEMA is not able to transfer DNA to bacteria no matter the variables. The data in Table 2 also indicate that [PMMA_3_-co-PDMAEMA_26_ (FRP)] obtained by FRP technique was more efficient to transform *E. coli* than the one obtained by RAFT (PMMA_31_-co-PDMAEMA_70_). These two copolymers differ by total molar mass, composition, and method of synthesis. Each one of these variables may be responsible for the difference in DNA transfer capability, as well as a combination of all. Molar mass is related to the size of the polyplex formed by combination with DNA, as well as toxicity (14,35-37). Differential composition is related to the hydrophilic/hydrophobic balance, which can certainly be a major factor to the interaction of the polyplex with the bacteria membrane. Finally, RAFT polymers present a sulphur group in one or both chain ends that can affect the DNA transfer process and toxicity. FRP polymers, on the other hand, present compositional inter-chain heterogeneity, making it difficult to know which chains specifically are the best transfer agent, composition-wise. All these scenarios are to be addressed in the future. We demonstrated here that copolymers of PDMAEMA and PMMA transferred DNA into *E. coli*, opening an opportunity to obtain more efficient and less toxic materials by varying their polymeric structure.

Transfer of foreign DNA into bacterial cells usually involves the generation of competence by a chemical treatment with CaCl_2_ followed by a heat shock perturbation (38). Alternatively, one may use electroporation, in which a strong and short electrical pulse is applied to perturb the lipid bilayer allowing for the penetration of charged molecules such as DNA (39). Although these techniques are well-established, the transformation of other prokaryotic cells such as *Leptospira*, a pathogenic bacterium of significant public health concern, or *Xanthomonas*, a phytopathogenic bacterium that infects economically relevant crops, is not straightforward. Hence, simpler and more efficient DNA delivery methods for prokaryotic organisms are desirable and will find applications under specific situations. Here, we showed that copolymers based on MMA and DMAEMA were able to deliver foreign DNA into *E. coli* cells.

The mechanism of gene delivery promoted by the copolymers tested here is unclear. However, the composition of the polymeric material was determinant to gene delivery efficiency. Hydrophilic polymers containing only DMAEMA were unable to deliver DNA into *E. coli* (Figure 5 and Table 2). The presence of MMA units was found to be crucial for the cases studied. In summary, amphiphilic copolymers containing DMAEMA and MMA units were shown to deliver DNA into *E. coli* cells and might open the way for the development of new DNA delivery agents, which could find a variety of technological applications.

## References

[1] Blanazs A, Armes SP, Ryan AJ (2009). Self-assembled block copolymer aggregates: from micelles to vesicles and their biological applications. Macromol Rapid Commun.

[2] Hu X, Zhang Y, Xie Z, Jing X, Bellotti A, Gu Z (2017). Stimuli-responsive polymersomes for biomedical applications. Biomacromolecules.

[3] Agut W, Brûlet A, Schatz C, Taton D, Lecommandoux S (2010). pH and temperature responsive polymeric micelles and polymersomes by self-assembly of poly2-(dimethylamino)ethyl methacrylate]-b-poly(glutamic acid) double hydrophilic block copolymers. Langmuir.

[4] Su Y, Dan M, Xiao X, Wang X, Zhang W (2013). A new thermo-responsive block copolymer with tunable upper critical solution temperature and lower critical solution temperature in the alcohol/water mixture. J Polym Sci Part A: Polym Chem.

[5] Kocak G, Tuncer C, Bütün V (2017). pH-responsive polymers. Polym Chem.

[6] de Souza JCP, Naves AF, Florenzano FH (2012). Specific thermo-responsiveness of PMMA-Block-PDMAEMA to selected ions and other factors in aqueous solution. Colloid Polym Sci.

[7] Ganta S, Devalapally H, Shahiwala A, Amiji M (2008). A review of stimuli-responsive nanocarriers for drug and gene delivery. J Control Release.

[8] Meng F, Zhong Z, Feijen J (2009). Stimuli-responsive polymersomes for programmed drug delivery. Biomacromolecules.

[9] Tanasienko IV, Yemets AI, Finiuk NS, Stoika RR, Blume YB (2015). DMAEM-based cationic polymers as novel carriers for DNA delivery into cells. Cell Biol Int.

[10] Mahato M, Kumar P, Sharma AK (2013). Amphiphilic polyethylenimine polymers mediate efficient delivery of DNA and SiRNA in mammalian cells. Mol Biosyst.

[11] Cheng C, Convertine AJ, Stayton PS, Bryers JD (2012). Multifunctional triblock copolymers for intracellular messenger RNA delivery. Biomaterials.

[12] Alhoranta AM, Lehtinen JK, Urtti AO, Butcher SJ, Aseyev VO, Tenhu HJ (2011). Cationic amphiphilic star and linear block copolymers: synthesis, self-assembly, and *in vitro* gene transfection. Biomacromolecules.

[13] Bai YX, Liu YB, Li YF, Zhang Q (2012). Synthesis and characterization of amphiphilic antibacterial copolymers. Polym Adv Technol.

[14] Sprouse D, Reineke TM (2014). Investigating the effects of block versus statistical glycopolycations containing primary and tertiary amines for plasmid DNA delivery. Biomacromolecules.

[15] Deshpande MC, Garnett MC, Vamvakaki M, Bailey L, Armes SP, Stolnik S (2002). Influence of polymer architecture on the structure of complexes formed by PEG-tertiary amine methacrylate copolymers and phosphorothioate oligonucleotide. J Control Release.

[16] Braunecker WA, Matyjaszewski K (2007). Controlled/living radical polymerization: features, developments, and perspectives. Progress Polym Sci.

[17] Perrier S (2017). 50th Anniversary Perspective: RAFT Polymerization - A User Guide. Macromolecules.

[18] Xiong Q, Ni P, Zhang F, Yu Z (2004). Synthesis and characterization of 2-(dimethylamino)ethyl methacrylate homopolymers via aqueous RAFT polymerization and their application in miniemulsion polymerization. Polym Bull.

[19] Franco C, Antonow MB, Beckenkamp A, Buffon A, Ceolin T, Tebaldi ML (2017). Data of PCL-b-P(MMA-DMAEMA)_2_ characterization and related assays. Data Brief.

[20] Sahnoun M, Charreyre MT, Veron L, Delair T, D'Agosto F (2005). Synthetic and characterization aspects of dimethylaminoethyl methacrylate reversible addition fragmentation chain transfer (RAFT) polymerization. J Polym Sci.

[21] Themistou E, Patrickios CS (2007). Synthesis and characterization of amphiphilic star copolymers of 2-(dimethylamino)ethyl methacrylate and methyl methacrylate: effects of architecture and composition. Eur Polym J.

[22] Singh M, Yadav A, Ma X, Amoah E (2010). Plasmid DNA transformation in *Escherichia coli*: effect of heat shock temperature, duration, and cold incubation of CaCl_2_ treated cells. Int J Biotechnol Biochem.

[23] Green R, Rogers EJ (2013). Transformation of chemically competent *E. coli.*. Methods Enzymol.

[24] Promega Corporation (2014). E. coli Competent Cells Protocol Technical Bulletin.

[25] Bergmans HE, van Die IM, Hoekstra WP (1981). Transformation in *Escherichia coli*: stages in the process. J Bacteriol.

[26] QIAGEN Plasmid Purification Handbook 06/2020 https://www.qiagen.com.

[27] Sambrook J, Russell DW  (2001). Molecular cloning: A laboratory manual.

[28] Xue Y, Wei D, Zheng A, Guan Y, Xiao H (2014). Study of stimuli-sensitivities of amphiphilic modified star poly [N, N-(dimethylamino) ethyl methacrylate] and its ability of DNA complexation. J Macromol Sci Part A, Pure and Appl Chem.

[29] Arigita C, Zuidam NJ, Crommelin DJA, Hennink WE (1999). Association and dissociation characteristic of polymer/DNA complexes used for gene delivery. Pharm Res.

[30] Ali U, Karim KJBA, Buang NA (2015). A review of the properties and applications of poly (methyl methacrylate) (PMMA). Polym Rev.

[31] Hollick EJ, Spalton DJ, Ursell PG, Pande MV (1998). Biocompatibility of poly(methyl methacrylate), silicone, and AcrySof intraocular lenses: Randomized comparison of the cellular reaction on the anterior lens surface. J Cataract Refract Surg.

[32] Neira-Velázquez MG, Rodríguez-Hernández MT, Hernández-Hernández E, Ruiz-Martínez ARY, Saldívar-Guerra E, Vivaldo-Lima E (2013). Polymer molecular weight measurement. Handbook of polymer synthesis, characterization, and processing.

[33] Yang Y, Liu L, Zhang J, Li C, Zhao H (2007). PMMA colloid particles stabilized by layered silicate with PMMA-b-PDMAEMA block copolymer brushes. Langmuir.

[34] Zhang YS (2016). Blue-white screening liquid can eliminate false positives in blue-white colony screening. Gene Mol Res.

[35] Cordeiro RA, Santo D, Farinha D, Serra A, Faneca H, Coelho JFJ (2017). High transfection efficiency promoted by tailor-made cationic tri-block copolymer-based nanoparticles. Acta Biomater.

[36] Fischer D, Bieber T, Li Y, Elsässer HP, Kissel T (1999). A novel non-viral vector for DNA delivery based on low molecular weight, branched polyethylenimine: effect of molecular weight on transfection efficiency and cytotoxicity. Pharm Res.

[37] Islam MA, Park TE, Singh B, Maharjan S, Firdous J, Cho MH (2014). Major degradable polycations as carriers for DNA and siRNA. J Control Release.

[38] Rahimzadeh M, Sadeghizadeh M, Najafi F, Arab S, Mobasheri H (2016). Impact of heat shock step on bacterial transformation efficiency. Mol Biol Res Commun.

[39] Potter H, Heller R (2018). Transfection by electroporation. Curr Protoc Mol Biol.

